# Neutrophil to High‐Density Lipoprotein Cholesterol Ratio and All‐Cause Mortality Among Asthmatic Individuals: A Cohort Study

**DOI:** 10.1155/mi/1427581

**Published:** 2026-08-29

**Authors:** Dong Li, Chengling Luo, Jing Xia

**Affiliations:** ^1^ Department of Thoracic Surgery, The Third Affiliated Hospital of Chongqing Medical University, Chongqing Medical University, Chongqing, China, cqmu.edu.cn; ^2^ Department of Respiratory and Critical Care Medicine, The Third Affiliated Hospital of Chongqing Medical University, Chongqing Medical University, Chongqing, China, cqmu.edu.cn

**Keywords:** asthma, mortality, neutrophil to high-density lipoprotein cholesterol ratio (NHR), predictive model, shapley additive explanations (SHAP)

## Abstract

**Background:**

At present, there is no epidemiological study available to confirm the efficacy of neutrophil to high‐density lipoprotein cholesterol ratio (NHR) in assessing the prognosis of the asthma population. This study aims to investigate the value of NHR in assessing the prognosis of asthma patients by utilizing data from National Health and Nutrition Examination Survey (NHANES) and constructing the predictive models.

**Methods:**

This study used Cox regression models, cumulative risk curves, and survival 3D interaction plots to check how NHR related to the outcomes for asthma patients. This study also used the least absolute shrinkage and selection operator (LASSO) regression screening to construct key variables for the prediction model, followed by the use of time‐dependent receiver operating characteristic (ROC) curves and Shapley additive explanations (SHAP) models to evaluate the performance and practical value of the prediction model.

**Results:**

The Cox regression models (HR: 1.12, 95% CI: 1.03–1.22), cumulative risk curves, and survival 3D interaction plots all confirmed that, after accounting for other factors, a higher NHR is linked to a lower survival rate and a higher risk of death for asthma patients. According to the LASSO regression and SHAP model, the five most important significant indicators predicting mortality in individuals with asthma were age, cholesterol, NHR, cardiovascular disease (CVD), and hypertension. The combination of these significant indicators produced superior performance when predicting the 1‐year (AUC: 0.874), 5‐year (AUC: 0.853), and 9‐year (AUC: 0.877) all‐cause mortality of asthma populations.

**Conclusions:**

In this nationally representative cohort of participants with asthma, elevated NHR was independently associated with higher all‐cause mortality risk. NHR may serve as a readily available marker reflecting systemic inflammatory‐metabolic status, but its clinical utility for asthma risk stratification requires further validation in independent cohorts.

## 1. Introduction

Chronic respiratory diseases are among the most prevalent causes of morbidity and mortality on a global scale. Asthma and chronic obstructive pulmonary disease (COPD) are the most prevalent chronic disorders that affect the respiratory system. Although these conditions are a major contribution to the world’s burden of noncommunicable disorders, they have received a lot less attention than diseases like cancer, diabetes, and heart disease [1]. The primary symptom of asthma is the recurrence of bronchospasm and respiratory inflammation, which in turn induce a range of symptoms, including dyspnea, chest constriction, wheezing, cough, and sputum [2, 3]. At present, it is affecting well over 350 million individuals worldwide, and the number of patients has been increasing over the past few decades. Each year, asthma results in the direct deaths of 250,000 individuals [4, 5]. Asthma is the second leading cause of death from chronic respiratory conditions worldwide [1]. So, finding a simple, reliable, and cost‐effective biomarker would have a big effect on how quickly treatment plans are made, how well preventative measures are put in place, and how early asthma is found.

Asthma is marked by airway inflammation, which plays a pivotal role in its advancement [6, 7]. Both localized and systemic inflammatory responses can intensify exacerbations of this disorder [8]. Although eosinophilic inflammation is traditionally emphasized in asthma, neutrophilic airway inflammation is increasingly recognized, particularly in severe, steroid‐resistant, or late‐onset asthma phenotypes [9, 10]. Research suggests that the neutrophil‐to‐lymphocyte ratio (NLR) may be linked to survival outcomes in asthma patients [11], indicating that neutrophilic inflammation is associated with poorer asthma control and worse prognosis.

High‐density lipoprotein cholesterol (HDL‐C) is a complex of proteins and low‐molecular‐weight, high‐density lipids [12], which has been shown to exert anti‐inflammatory and antioxidant effects within the lung microenvironment [13, 14]. HDL particles participate in a range of physiological processes, including innate immunity, protease activity regulation, thrombosis modulation, and inflammatory response mediation [15]. Epidemiological evidence from large‐scale cohort studies has consistently identified HDL‐C as a valuable predictor of coronary heart disease risk [16]. However, recent investigations uncovered the U‐shaped link between HDL‐C concentrations and cardiovascular disease (CVD) mortality [17]. Additionally, some studies have shown that very low HDL‐C levels are strongly connected to a higher risk of dying from cancer [18, 19], and another study found that HDL‐C levels are also linked to death from pulmonary fibrosis [20].

Recognizing the diverse beneficial roles of HDL in the human body, researchers have investigated the clinical relevance of HDL‐C‐associated inflammatory biomarkers. Recently, neutrophil to HDL‐C ratio (NHR) has gained attention as an emerging biomarker. It can be readily derived and measured using standard blood test data [21]. Some studies have shown the link between NHR and prognosis of depression, kidney stones, periodontitis, and frailty in people [22–26]. However, the relationship between the NHR and the prognosis of asthma patients is still uncertain.

It is hypothesized that the NHR may be linked to asthma prognosis and may give a more complete picture of systemic inflammatory status than neutrophil or HDL‐C alone. Utilizing information from National Health and Nutrition Examination Survey (NHANES) 2011–2018, the study aims to investigate the relationships between NHR and mortality risk among individuals with asthma.

## 2. Materials and Methods

### 2.1. Study Data and Population

All data of the study were extracted from the NHANES, maintained by the Centers for Disease Control and Prevention. All information obtained from NHANES was anonymized to ensure participant confidentiality. NHANES data are publicly available and fully de‐identified in accordance with the Health Insurance Portability and Accountability Act (HIPAA) standards to ensure participant privacy and confidentiality. As such, this secondary analysis of anonymized, publicly accessible data did not require additional institutional review board approval. This investigation analyzed NHANES data from 2011 to 2018, obtained from https://www.cdc.gov/nchs/nhanes/index.htm. After excluding individuals with missing follow‐up data, asthma diagnosis, NHR values, or covariates, the final analytical cohort comprised 2719 asthmatic participants (eFigure 1).

### 2.2. Independent and Outcome Variables

The primary variables examined in this study were the NHR and mortality. NHR was defined as ratios of blood neutrophil counts to HDL‐C levels. To assess the follow‐up status of study participants, we utilized death index data up to December 31, 2019. The NCHS offered detailed insights into the matching methodology employed. Mortality status was determined using ICD‐10.

### 2.3. Confounding Variable

To address the influence of potential confounding factors, our study incorporated a comprehensive set of covariates. These included demographic characteristics (sex, race, and age), socioeconomic factors (education level and marital status), lifestyle habits (smoking and alcohol), physical examinations (BMI), medication use (glucocorticoids [GCS]), and medical conditions (hay fever, CVD, hypertension, diabetes, other chronic airway disease [CAD] except asthma, and cancer). Additionally, we accounted for laboratory parameters such as serum total cholesterol (STC), blood eosinophil count, and serum triglycerides (STG). This research utilized a standard medical questionnaire to identify individuals with asthma. Has a medical professional diagnosed you with asthma? Affirmative responses suggested a diagnosis of asthma.

### 2.4. Statistical Analysis

Statistical analyses were carried out using the chi‐square test for categorical information and the Kruskal–Wallis rank‐sum test for continuous information. To examine the association between mortality risk in asthmatics and NHR, three Cox proportional hazards regression models were employed. To assess the linear trend across NHR tertiles, we conducted trend tests by assigning the median value of each tertile to its corresponding group and modeling this as a continuous variable in the Cox regression models. The *p* for trend was derived from this model to evaluate whether there was a linear relationship between NHR and mortality. RCS was then utilized to further quantify this relationship, adjusting for all covariates. The cumulative death risk curve, complemented by the survival 3D plot [27], quantified the impact of NHR on survival outcomes throughout the follow‐up period. In addition, the predictive model and Shapley additive explanations (SHAP) model were constructed by employing the least absolute shrinkage and selection operator (LASSO) regression to select key variables. This approach enhanced the prediction precision of the model and safeguards against overfitting. The SHAP model was subsequently implied to determine the corresponding importance of all variables in predicting mortality risk among asthma patients. To reduce the risk of overestimating model performance, we performed internal validation using 1000 bootstrap resamples and 10‐fold cross‐validation. Bootstrap validation was used to evaluate the stability of time‐dependent AUC estimates, and 10‐fold cross‐validation was used to assess the discriminatory performance of the model across resampled datasets. Time‐dependent AUC values were calculated for 1‐, 5‐, and 9‐year all‐cause mortality. Missing covariates, each constituting less than 10% of the total data, were managed through multiple imputations [28]. Sensitivity analyses were conducted on the primary findings using the imputed dataset. Appropriate weights were applied to ensure sample representativeness. This study applied R 4.4.1 to carry out a series of analyses. The *p* less than 0.05 implied a statistical difference. A more detailed description is provided in the Supporting Information.

## 3. Results

### 3.1. Comparison of Baseline Information

This study included 2719 participants with asthma, whose characteristics were grouped by NHR tertiles (eTable 1). The statistical difference was discovered across various NHR groups in terms of smoke status, sex, education level, race, other CAD history, diabetes, hypertension, CVD history, survival status, BMI, alcohol consumption, eosinophil, STC, and STG. The likelihood of asthma patients dying increased as the NHR increased.

### 3.2. Association Between NHR and Mortality

**Figure 1:**
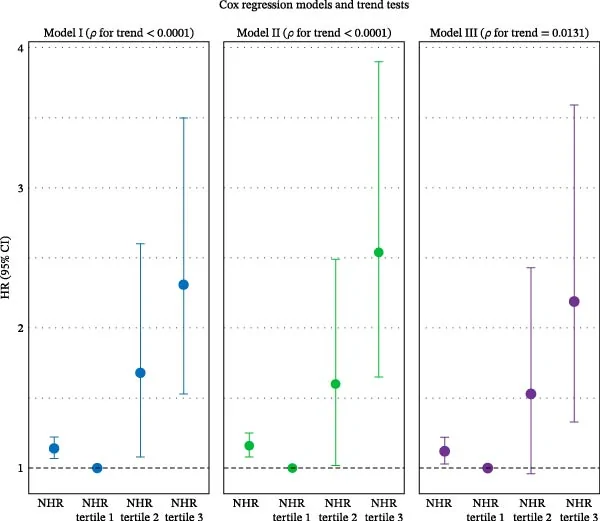
Association between NHR and all‐cause mortality among participants with asthma. Hazard ratios were estimated using Cox proportional hazards regression models with NHR modeled as tertiles. Model I was unadjusted. Model II was adjusted for age, race, and sex. Model III was adjusted for all covariates. The tertile ranges were 0.19–2.51 for Tertile 1, 2.52–3.99 for Tertile 2, and 4.00–16.11 for Tertile 3. NHR, neutrophil‐to‐high‐density lipoprotein cholesterol ratio.

**Figure 2:**
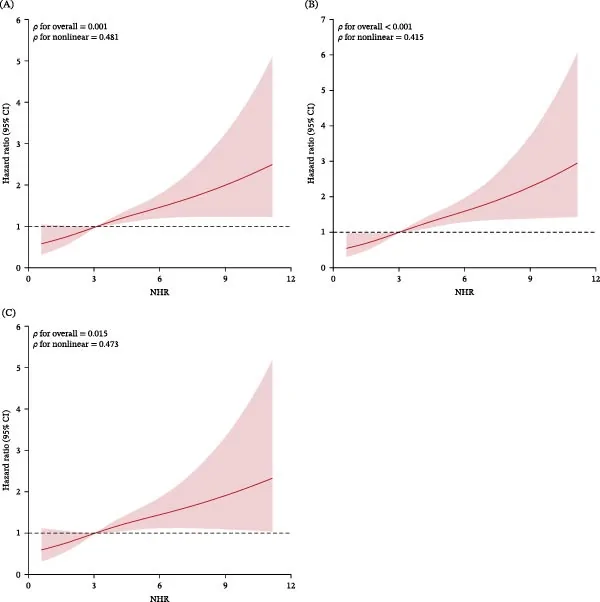
Dose‐response association between NHR and all‐cause mortality among participants with asthma. RCS analyses were performed using Model I (A), Model II (B), and Model III (C). The solid line represents the estimated hazard ratio, and the shaded area represents the 95% confidence interval.

This study exploited three Cox regression analyses to quantify the link between NHR with mortality risk in asthma patients (Figure 1). In model III, after adjusting for all confounding variables, NHR was related to elevated mortality risk. Specifically, each one‐unit increase in NHR was associated with a 12% higher risk of all‐cause death (HR: 1.12, 95% CI: 1.03–1.22; *p* = 0.0101). When NHR was analyzed by tertiles, participants in the highest tertile (T3: 4.00–16.11) had a significantly higher death risk than those in the lowest tertile (T1: 0.19–2.51; HR: 2.19, 95% CI: 1.33–3.59; *p* = 0.0020). The association for the middle tertile was not statistically significant in model III (HR: 1.53, 95% CI: 0.96–2.43; *p* = 0.0749), whereas the overall trend across increasing NHR tertiles remained significant (*p* for trend = 0.0017) (Figure 1). These findings were consistent with the RCS analysis, which supported a positive and linear association between NHR and all‐cause mortality, with *p* for overall < 0.05 and *p* for nonlinear > 0.05 (Figure 2).

### 3.3. Cumulative Death Risk Curve and Survival 3D Plot

**Figure 3:**
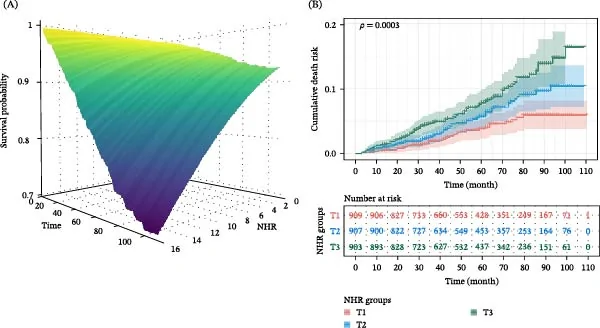
Association of NHR with survival probability and cumulative mortality among participants with asthma. (A) Survival 3D plot showing the relationship among NHR, follow‐up time, and survival probability. (B) Cumulative mortality curve according to NHR level.

This study also used the survival 3D plot (Figure 3A) and the cumulative death risk curve (Figure 3B) to identify the impact of NHR on outcomes among asthma patients. Both models suggested that the larger the NHR, the lower the survival probability and higher the death risk for asthma patients.

### 3.4. Constructing Predictive Models

**Figure 4:**
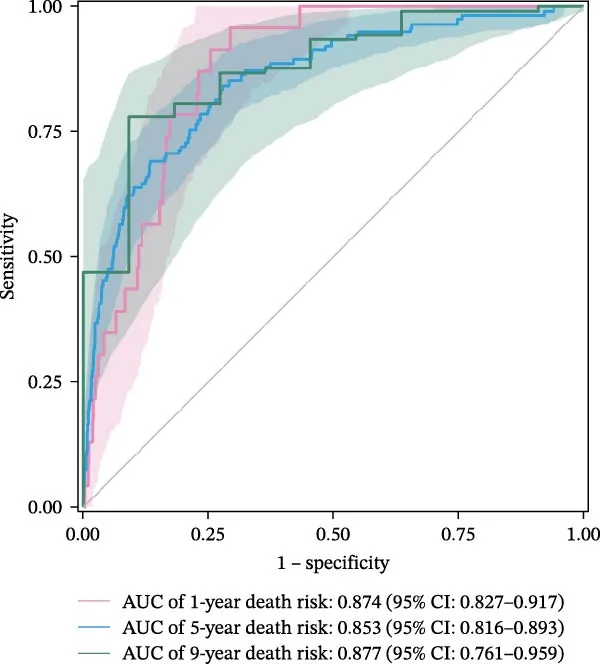
Time‐dependent ROC curves for the exploratory prognostic model and boosting validation. Time‐dependent ROC curves were used to assess the discriminatory performance of the model for predicting 1‐, 5‐, and 9‐year all‐cause mortality among participants with asthma. Internal validation was performed using bootstrap resampling.

**Figure 5:**
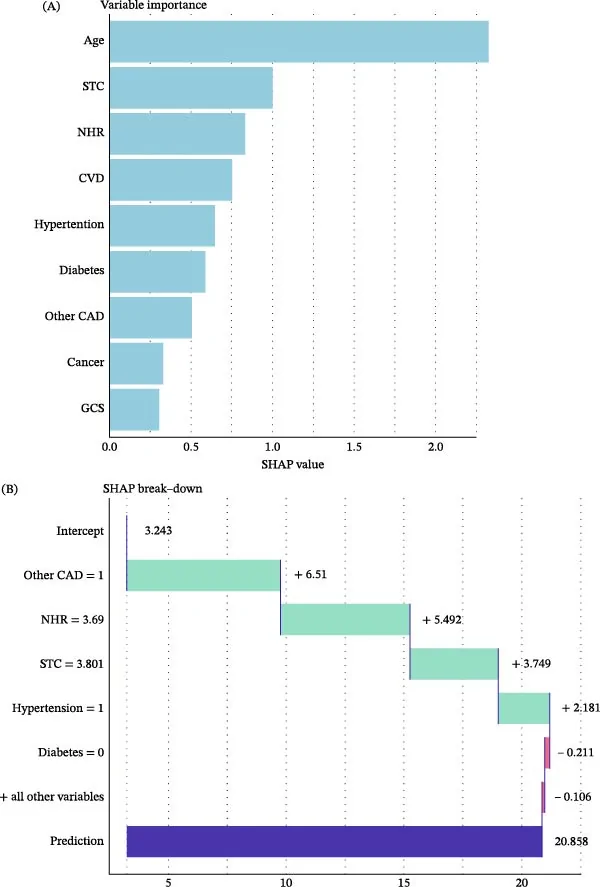
SHAP‐based interpretation of the exploratory prognostic model. (A) SHAP summary plot showing the relative importance of the nine selected predictors for all‐cause mortality. (B) Individual‐level SHAP explanation illustrating the contribution of each predictor to the estimated mortality risk in a representative participant with asthma. SHAP, SHapley Additive exPlanations.

To identify the relevant variables required for the development of a predictive model, the study implemented Lasso regression. eFigure 2 illustrated the variable coefficient characteristics. Use the 10‐fold cross‐validation method for iterative analysis. NHR, age, hypertension, diabetes, CVD history, cancer history, other CAD history, GCS, and STC were the nine variables that were closely associated with the prognosis of asthma patients. The combination of these nine significant indicators produced superior performance (Figure 4) when predicting the 1‐year (AUC: 0.874), 5‐year (AUC: 0.853), and 9‐year (AUC: 0.877) all‐cause mortality of asthma populations. In the apparent model, the AUC values for predicting 1‐, 5‐, and 9‐year all‐cause mortality were 0.874, 0.853, and 0.877, respectively. To assess the robustness of the model performance, we further performed internal validation using bootstrap resampling and cross‐validation. In bootstrap validation, the bootstrap‐derived AUC ranges were 0.827–0.917 for 1‐year mortality, 0.816–0.893 for 5‐year mortality, and 0.761–0.959 for 9‐year mortality. In cross‐validation, the validated AUC values were 0.868, 0.845, and 0.872 for 1‐, 5‐, and 9‐year mortality, respectively (eTable 2). These findings indicated that the exploratory prognostic model retained acceptable discriminatory performance after internal validation. Subsequently, the relative contribution of the nine selected predictors was estimated for the interpretability of the exploratory prognostic model using SHAP analysis (Figure 5A). SHAP summary plot showed that age, STC, NHR, CVD, and hypertension were the top five predictors for all‐cause mortality in participants with asthma. The individual‐level SHAP explanation also revealed the positive contribution of selected variables such as other CAD, NHR, STC, and hypertension to the estimated mortality risk in a representative participant (Figure 5B). These findings suggest that the NHR contributed to model prediction together with established demographic and clinical risk factors. However, SHAP analysis was used for model interpretation and should not be interpreted as defining clinical thresholds or treatment decision rules.

### 3.5. Subgroup Analysis and Sensitive Analysis

The subgroup analysis (eTable 3) showed that NHR was related to the higher mortality risk in white women over 60 with a BMI of 25 or more who had high blood pressure but no hay fever, diabetes, CVD, other CAD, cancer, or usage of GCS. Additionally, conduct sensitivity studies on the primary outcomes using data from multiple imputations. Following multiple imputations for missing covariates, the outcomes from the imputed data remained consistent with the primary analysis: the growing NHR was related to incremental mortality risk (eTable 4 and eFigure 3). To further contextualize the prognostic relevance of NHR, we compared its discriminatory performance with that of the NLR, an established systemic inflammatory marker. In time‐dependent receiver operating characteristic (ROC) analysis, the AUC values of NHR for predicting 1‐, 5‐, and 9‐year all‐cause mortality were 0.71, 0.67, and 0.66, respectively (eFigure 4). The corresponding AUC values of NLR were 0.62, 0.58, and 0.61, respectively. These findings indicate that NHR showed better discriminatory performance than NLR for mortality risk prediction among participants with asthma, although the predictive ability of NHR alone remained moderate.

## 4. Discussion

This study was a large‐scale cohort study to identify the association between NHR and mortality in asthma participants. This study showed that increasing NHR was linked with elevated mortality in people with asthma. The LASSO regression and SHAP model indicated that the five most important significant indicators predicting mortality in individuals with asthma were age, STC, NHR, CVD, and hypertension. The combination of these significant indicators produced superior performance when predicting the 1‐year (AUC: 0.874), 5‐year (AUC: 0.853), and 9‐year (AUC: 0.877) survival rates of asthma populations.

Recently, investigations demonstrated that NHR, monocyte‐high‐density lipoprotein ratio (MHR), et cetera, may become emerging inflammatory markers for predicting the onset and prognosis of many diseases, owing to their simplicity and accessibility [22–26, 29]. Epidemiological studies identified a positive association between the NHR and conditions such as periodontitis [22], gallstone disease [24], and depression risk [23], suggesting the NHR’s potential as an inflammatory biomarker for these disorders. Another investigation demonstrated that elevated NHR independently predicts all‐cause and CVD mortality among ordinary populations [25]. An observational study found that there was a J‐shaped relationship between NHR and frailty among American adults [26]. Researchers also applied NHR to predict long‐term death in frail populations. Furthermore, studies on COVID‐19 patients who were critically ill showed lower HDL‐C levels and higher NHR values at admission compared to those of individuals who were not critically sick. These factors were strongly associated with a poorer prognosis, particularly in subgroups with COVID‐19 and diabetes [30].

Neutrophils are the main effector cells of the innate immune system and play a crucial role in the pathophysiology of asthma. Neutrophilia has been strongly linked to increased asthma severity, corticosteroid resistance, and a higher frequency of acute exacerbations [31]. By releasing reactive oxygen species (ROS), neutrophil extracellular traps (NETs), and proteolytic enzymes such as myeloperoxidase and elastase, neutrophils exacerbate airway inflammation and contribute to oxidative stress [32–34]. These processes promote a vicious cycle of inflammation, tissue injury, and airway remodeling in severe asthma, ultimately leading to poor disease control, worsened clinical outcomes, and an increased risk of mortality [35].

In contrast, HDL‐C exerts multiple protective effects beyond its role in lipid transport. It has demonstrated anti‐inflammatory, antioxidant, anti‐infective, and antithrombotic properties. HDL‐C can stop neutrophils from becoming active, lower the amount of ROS produced, limit the creation of NETs, and block nuclear factor kappa‐light‐chain‐enhancer of activated B cells (NF‐κB) signaling pathways [36–38]. Decreased HDL‐C levels are frequently observed in chronic inflammatory diseases and are associated with heightened systemic inflammation and oxidative stress. In asthma, an inverse association has been reported between HDL‐C levels and eosinophil counts [39], further supporting its immunomodulatory role.

One cohort research discovered that an incremental MHR was related to an incremental mortality risk among people with asthma [29]. Similarly, several investigations have identified that incremental immunoinflammatory biomarkers, such as NHR and MHR, are related to the occurrence of major adverse CVD events and that the combination of these indicators can more accurately predict the likelihood of major adverse cardiovascular events (MACE) in patients with nonobstructive coronary myocardial infarction [40]. NHR perhaps serves as a more reliable biomarker than HDL‐C and is an important measure of inflammation and oxidative stress status, as well as having an independent predictive value for the development of CVD, Parkinson’s disease, depression, and other disorders [41, 42]. Moreover, it has been shown that the NHR may be useful in predicting thrombosis in patients with true erythrocytosis [43]. HDL‐C stops macrophages from moving around in atherosclerosis and makes it easier for oxidized low‐density lipoprotein cholesterol to be removed from these cells [44]. The substance has anti‐inflammatory and lipid‐regulating effects [44].

NHR combines both substances that cause inflammation and those that reduce it, acting as a combined indicator of problems in the immune system and oxidative stress. Elevated NHR levels may therefore reflect a heightened inflammatory burden, contributing to poor clinical outcomes in individuals with asthma. This hypothesis is supported by prior studies demonstrating associations between high NHR and adverse outcomes in CVD and gallstone diseases [24, 40]. Given its simplicity, low cost, and clinical accessibility, NHR holds promise as a novel biomarker for prognostic assessment and risk stratification in asthma. Further experimental and longitudinal studies are warranted to clarify the underlying biological mechanisms and to validate its clinical utility.

Unlike previous studies, this study is designed to clarify the association of NHR with mortality among asthmatic people, which has not been previously reported by others. Utilizing a large, nationally representative sample, we adjusted for numerous potential confounders to strengthen the validity of our findings. The SHAP model quantified the relative importance of variables in predicting mortality, emphasizing the NHR’s significance. Also, the linear positive association between NHR and mortality stayed the same after multiple imputations were used to fill in the missing data. This shows that our results are strong. NHR’s simplicity and availability enhance its real‐world applicability, particularly in resource‐limited settings where complex multimarker panels may be impractical. These findings offer different perspectives on asthma management and treatment, suggesting new directions for future research.

Despite these strengths, several limitations should be considered. First, asthma was identified on the basis of self‐reported physician diagnosis rather than objective diagnostic testing or clinical adjudication. We, therefore, could not reliably distinguish current asthma from a historical diagnosis, asthma from COPD, or asthma‐COPD overlap. This may have introduced diagnostic misclassification and increased clinical heterogeneity, potentially affecting the estimated association between NHR and all‐cause mortality. Accordingly, our findings should be interpreted as applying to individuals with self‐reported physician‐diagnosed asthma rather than to a cohort with objectively confirmed asthma. Second, several clinically important asthma‐related variables were unavailable, including disease severity, inhaled corticosteroid use, exacerbation frequency, and longitudinal treatment changes. Although glucocorticoid use during the preceding month was included as a covariate, this measure was relatively broad and could not capture asthma‐specific treatment intensity or cumulative corticosteroid exposure. Residual confounding related to disease severity and treatment therefore cannot be excluded. Third, NHR is a nonspecific marker of systemic inflammatory and lipid‐related immune status rather than an asthma‐specific biomarker. Neutrophil counts and HDL‐C levels may be influenced by acute infection, systemic inflammatory conditions, recent asthma exacerbations, corticosteroid exposure, and other medications. Detailed information on acute clinical conditions at the time of blood collection was not available, and NHR was measured only once at baseline. Consequently, a single measurement may not fully reflect long‐term inflammatory‐metabolic status, and residual confounding from transient inflammatory states remains possible. Fourth, the primary outcome was all‐cause mortality. We did not distinguish asthma‐related deaths from cardiovascular, malignant, infectious, metabolic, or other respiratory causes. The observed association may therefore reflect broader systemic inflammatory and comorbidity‐related risks rather than asthma‐specific mortality alone. Finally, although bootstrap validation and cross‐validation supported the internal stability of the exploratory prognostic model, external validation was not available. Its generalizability to independent asthma populations, different healthcare settings, and other ethnic groups therefore remains uncertain. Future prospective studies incorporating objective asthma diagnosis, detailed clinical phenotyping, repeated NHR measurements, cause‐specific mortality assessment, and external model validation are warranted.

## 5. Conclusion

In this nationally representative cohort of participants with asthma, elevated NHR was independently associated with an increased all‐cause mortality risk. NHR may serve as an accessible marker reflecting systemic inflammatory‐metabolic status and may provide soupprting information for mortality risk assessment when considered alongside established clinical risk factors. However, given the modest effect size, the nonspecific nature of NHR, and the lack of external validation, further prospective studies are needed before NHR can be incorporated into routine asthma risk stratification or clinical decision‐making.

## Author Contributions

Conceptualization: Jing Xia, Chengling Luo, and Dong Li. Data collection, statistical analysis: Jing Xia and Dong Li. Original draft: Dong Li. Review and editing: Jing Xia and Chengling Luo.

## Funding

No funding was received for this manuscript.

## Disclosure

All authors have read and approved the final manuscript for publication.

## Ethics Statement

The Research Ethics Review Board of the National Center for Health Statistics has approved all NHANES research protocols (Protocol #2011‐17 and Protocol #2018‐01). Written informed consent was obtained from all subjects.

## Conflicts of Interest

The authors declare no conflicts of interest.

## Supporting Information

Additional supporting information can be found online in the Supporting Information section.

## Supporting information


**Supporting Information** eTable 1: Baseline characteristics of participants with asthma according to NHR tertiles. eTable 2: Internal validation of the exploratory prognostic model using bootstrap resampling and cross‐validation. eTable 3: Subgroup analyses of the association between NHR and all‐cause mortality among participants with asthma. eTable 4: Sensitivity analysis of the association between NHR and all‐cause mortality after multiple imputation. eFigure 1: Flow chart of participant selection. eFigure 2: Variable selection using LASSO regression. (A) Cross‐validation plot for selecting the optimal penalty parameter. (B) Coefficient path plot showing variable selection across different penalty levels. LASSO, least absolute shrinkage and selection operator. eFigure 3: Association of NHR with survival probability and cumulative mortality after multiple imputation. (A) Survival 3D plot showing the relationship among NHR, follow‐up time, and survival probability. (B) Cumulative mortality curve according to NHR level. eFigure 4: Comparison of time‐dependent ROC curves between NHR and NLR for predicting all‐cause mortality among participants with asthma. Time‐dependent ROC curves were generated to compare the discriminatory performance of NHR and NLR for 1‐, 5‐, and 9‐year all‐cause mortality.

## Data Availability

Data are available upon request from the authors.
