# Supplementary material for: Neutrophil to High‐Density Lipoprotein Cholesterol Ratio and All‐Cause Mortality Among Asthmatic Individuals: A Cohort Study
Source: Mediators Inflamm. 2026 Aug 29;2026:1427581. doi: 10.1155/mi/1427581 (PMC13525437; doi:10.1155/mi/1427581)
Supplement: Supplementary file 1 [file MI-2026-1427581-s001.pdf]

eTable 1. Baseline characteristics of study populations based on NHR tertile groups.

	NHR Tertile 1 (0.19-2.51)	NHR Tertile 2 (2.52-3.99)	NHR Tertile 3 (4.00-16.11)	P value
Sex (%)				<0.0001
Female	65.36	60.15	52.81	
Male	34.64	39.85	47.19	
Race (%)				<0.0001
Other Race populations	16.38	20.39	22.36	
White populations	63.72	69.57	70.83	
Black populations	19.90	10.04	6.81	
Education (%)				0.0026
Less than high school	9.82	12.15	14.95	
High school	18.05	21.39	23.56	
More than high school	72.13	66.46	61.48	
Marriage (%)				0.7499
Married	49.28	50.73	52.50	
Single	43.69	41.55	39.74	
Living with a partner	7.03	7.73	7.76	
Smoke (%)				<0.0001
No	60.92	52.17	45.94	
Yes	39.08	47.83	54.06	

GCS (%)				0.1611
No	84.71	85.16	80.16	
Yes	15.29	14.84	19.84	
Hypertension (%)				0.0001
No	68.47	65.45	56.60	
Yes	31.53	34.55	43.40	
Diabetes (%)				<0.0001
No	95.25	88.46	80.67	
Yes	4.75	11.54	19.33	
CVD (%)				0.0009
No	90.95	87.80	83.56	
Yes	9.05	12.20	16.44	
Other CAD (%)				0.0278
No	78.64	79.10	72.15	
Yes	21.36	20.90	27.85	
Cancer (%)				0.5228
No	89.92	87.66	87.30	
Yes	10.08	12.34	12.70	
Hay fever (%)				0.1075
No	87.63	86.16	82.55	
Yes	12.37	13.84	17.45	

Status (%)				0.0003
Alive	97.25	95.62	93.43	
Death	2.75	4.38	6.57	
Age (years)	46.00 (30.00 ,61.00)	44.00 (29.00 ,61.00)	44.00 (31.00 ,58.00)	0.4944
BMI (kg/m2)	26.10 (22.80 ,30.40)	28.90 (24.90 ,34.10)	32.80 (27.70 ,39.50)	<0.0001
Alcohol intake (gm)	17.87±2.05	11.63±1.38	7.79±0.93	0.0004
Eosinophil (1000 cells/uL)	0.20±0.01	0.23±0.01	0.26±0.01	0.0002
STC (mmol/l)	5.09±0.05	4.93±0.07	4.86±0.04	0.0174
STG (mmol/l)	1.02 (0.72 ,1.40)	1.29 (0.95 ,1.96)	1.93 (1.24 ,2.79)	<0.0001

Note: Median and IQR for continuous variables with non-normal distributions; mean ± SD for continuous variables with normal distributions. Proportions were employed to describe categorical variables. SD: standards deviation; Cardiovascular disease: CVD; Chronic airway disease: CAD; Serum total cholesterol: STC; Serum triglycerides: STG; Neutrophil-high-density lipoprotein cholesterol ratio: NHR.

eTable 2. Internal validation of the exploratory prognostic model using bootstrap resampling and cross-validation.

Time point	Apparent AUC	Bootstrap-derived AUC range	Cross-validated AUC
1-year death risk	0.874	0.827–0.917	0.868
5-year death risk	0.853	0.816–0.893	0.845
9-year death risk	0.877	0.761–0.959	0.872

eTable 3. Subgroup analyses of the association between NHR and all-cause mortality among participants with asthma.

Subgroup	HR (95% CI)	P value
**Sex**		
Male	1.08 (0.97, 1.22)	0.1691
Female	1.22 (1.06, 1.40)	0.0044
**Age**		
<60	1.14 (0.98, 1.32)	0.0803
≥60	1.11 (1.00, 1.24)	0.0439
**Race**		
Other Race populations	1.20 (0.99, 1.46)	0.0661
White populations	1.15 (1.03, 1.29)	0.0103
Black populations	0.98 (0.78, 1.24)	0.8797
**BMI**		
<25	1.11 (0.89, 1.39)	0.3630
25-30	1.20 (1.00, 1.43)	0.0455
≥30	1.13 (1.00, 1.28)	0.0468
**GCS**		
No	1.13 (1.01, 1.26)	0.0335
Yes	1.10 (0.95, 1.27)	0.2223
**Hay fever**		

No	1.11 (1.01, 1.22)	0.0370
Yes	1.21 (0.97, 1.52)	0.0951
**Hypertension**		
No	1.11 (0.90, 1.38)	0.3328
Yes	1.15 (1.04, 1.27)	0.0049
**Diabetes**		
No	1.18 (1.05, 1.32)	0.0056
Yes	1.10 (0.95, 1.27)	0.2048
**CVD**		
No	1.15 (1.02, 1.29)	0.0198
Yes	1.07 (0.94, 1.22)	0.3048
**Other CAD**		
No	1.27 (1.13, 1.44)	0.0001
Yes	1.03 (0.92, 1.15)	0.6471
**Cancer**		
No	1.19 (1.08, 1.31)	0.0006
Yes	1.02 (0.86, 1.22)	0.8150

Note: All stratified analyses adjusted for all covariates except for the stratification variable.

eTable 4. Sensitivity analysis of the association between NHR and all-cause mortality after multiple imputation.

	**Model I**	**Model II**	**Model III**
	**HR (95% CI) P value**	**HR (95% CI) P value**	**HR (95% CI) P value**
NHR	1.14 (1.07, 1.22) <0.0001	1.15 (1.08, 1.23) <0.0001	1.11 (1.02, 1.20) 0.0145
NHR tertile groups			
NHR Tertile 1	Reference	Reference	Reference
NHR Tertile 2	1.40 (0.94, 2.09) 0.0992	1.35 (0.90, 2.03) 0.1476	1.31 (0.86, 2.01) 0.2040
NHR Tertile 3	2.28 (1.58, 3.29) <0.0001	2.31 (1.58, 3.38) <0.0001	1.92 (1.24, 2.98) 0.0034
**P for trend**	<0.0001	<0.0001	0.0025

Note: Model I adjusted for none. Model II adjusted for age, race, and sex. Model III adjusted for all covariates (race, age, sex, smoking, alcohol, education, marriage, BMI, GCS, hypertension, diabetes, CVD, cancer, other CAD, hay fever, STC, STG and blood eosinophil counts).

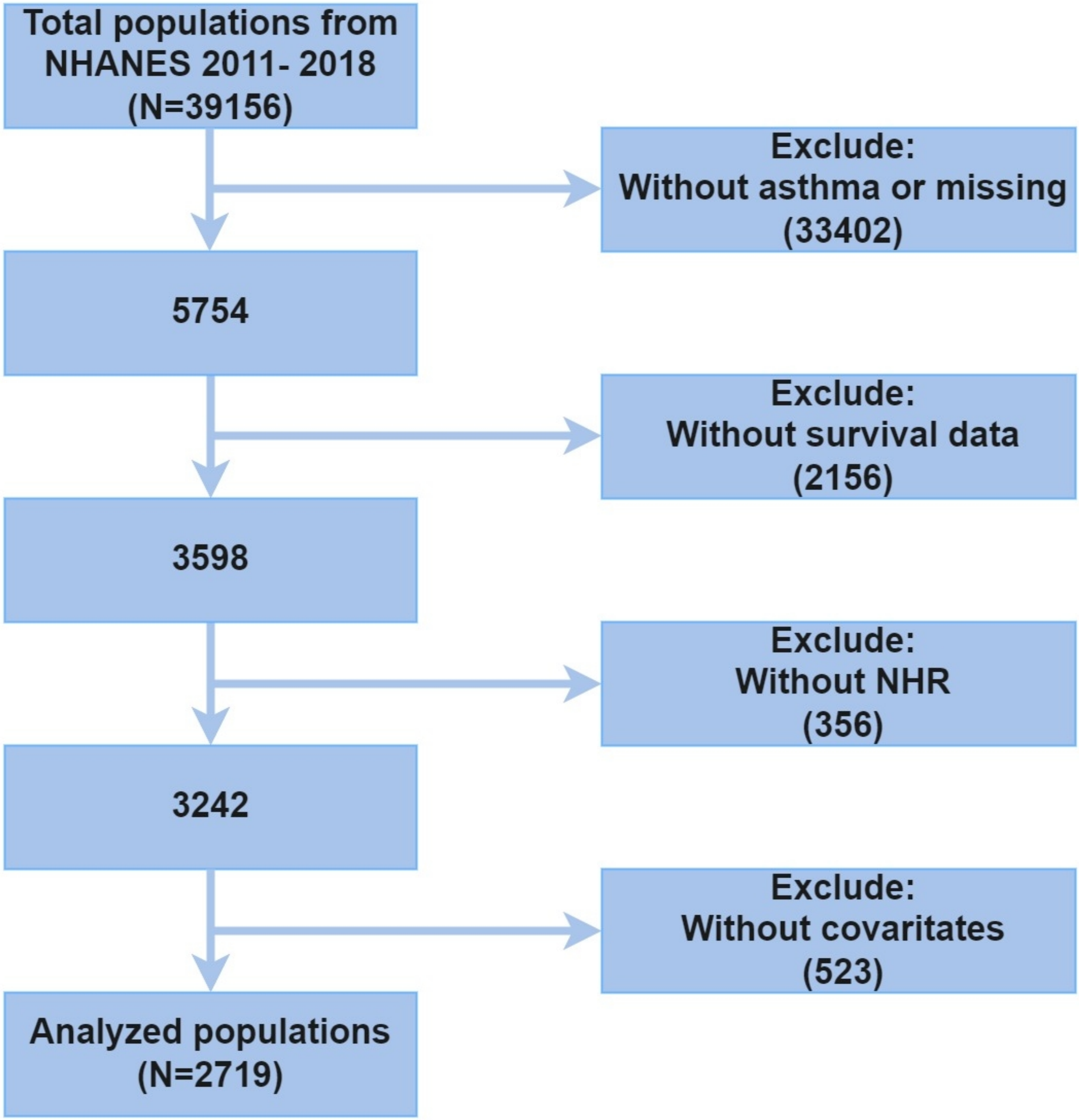

eFigure 1. Flow chart of participant selection.

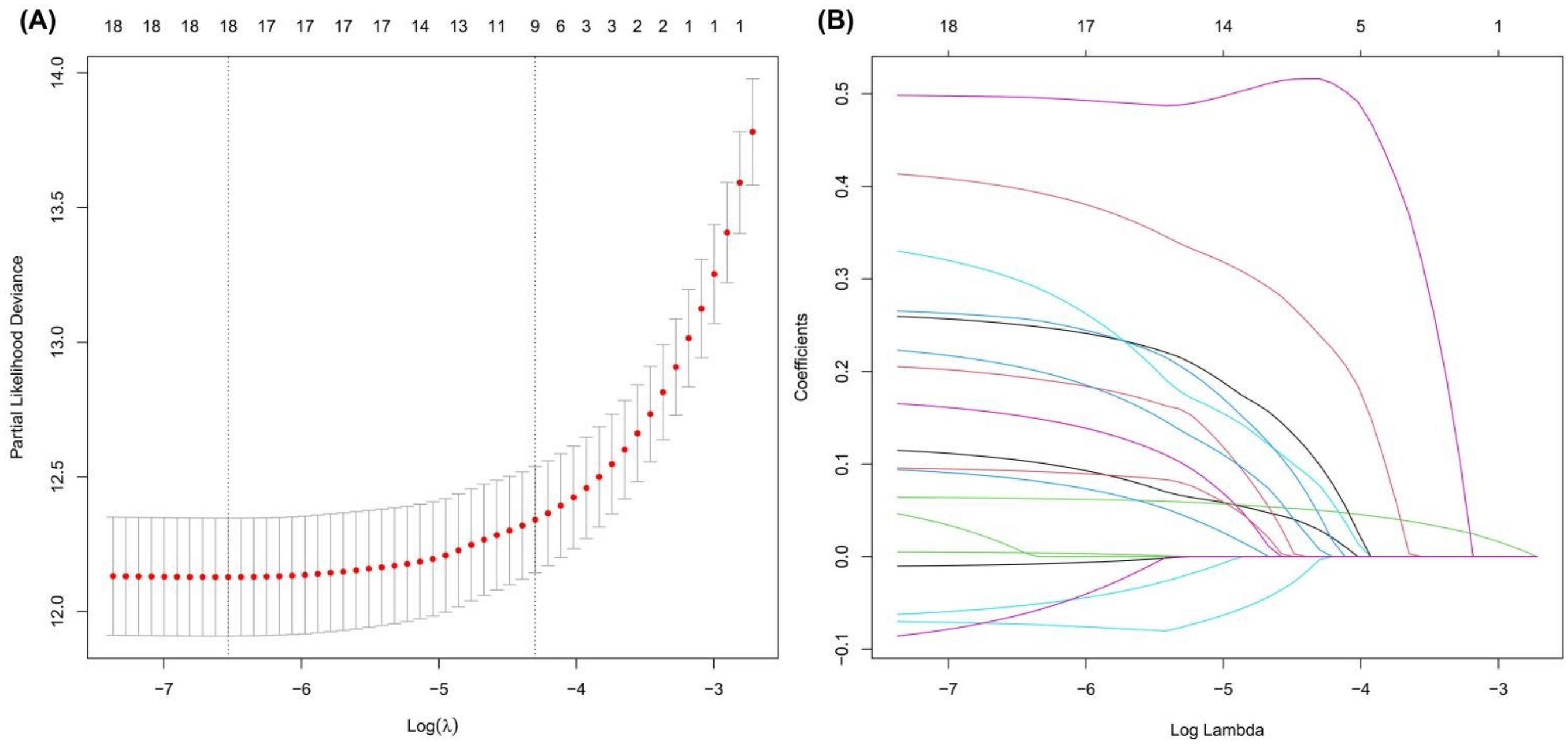

eFigure 2. Variable selection using LASSO regression.

(A) Cross-validation plot for selecting the optimal penalty parameter. (B) Coefficient path plot showing variable selection across different penalty levels. LASSO, least absolute shrinkage and selection operator.

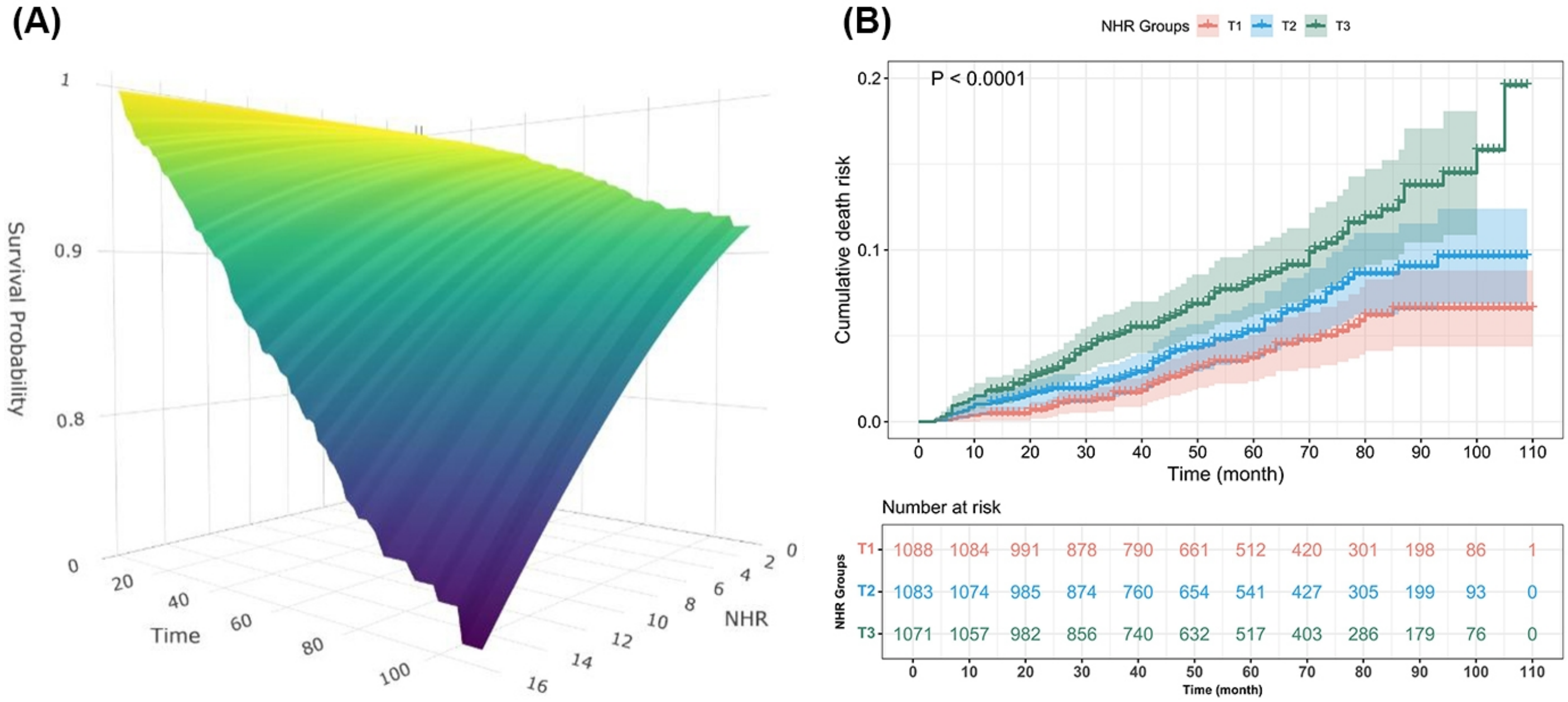

eFigure 3. Association of NHR with survival probability and cumulative mortality after multiple imputation.

(A) Survival 3D plot showing the relationship among NHR, follow-up time, and survival probability. (B) Cumulative mortality curve according to NHR level.

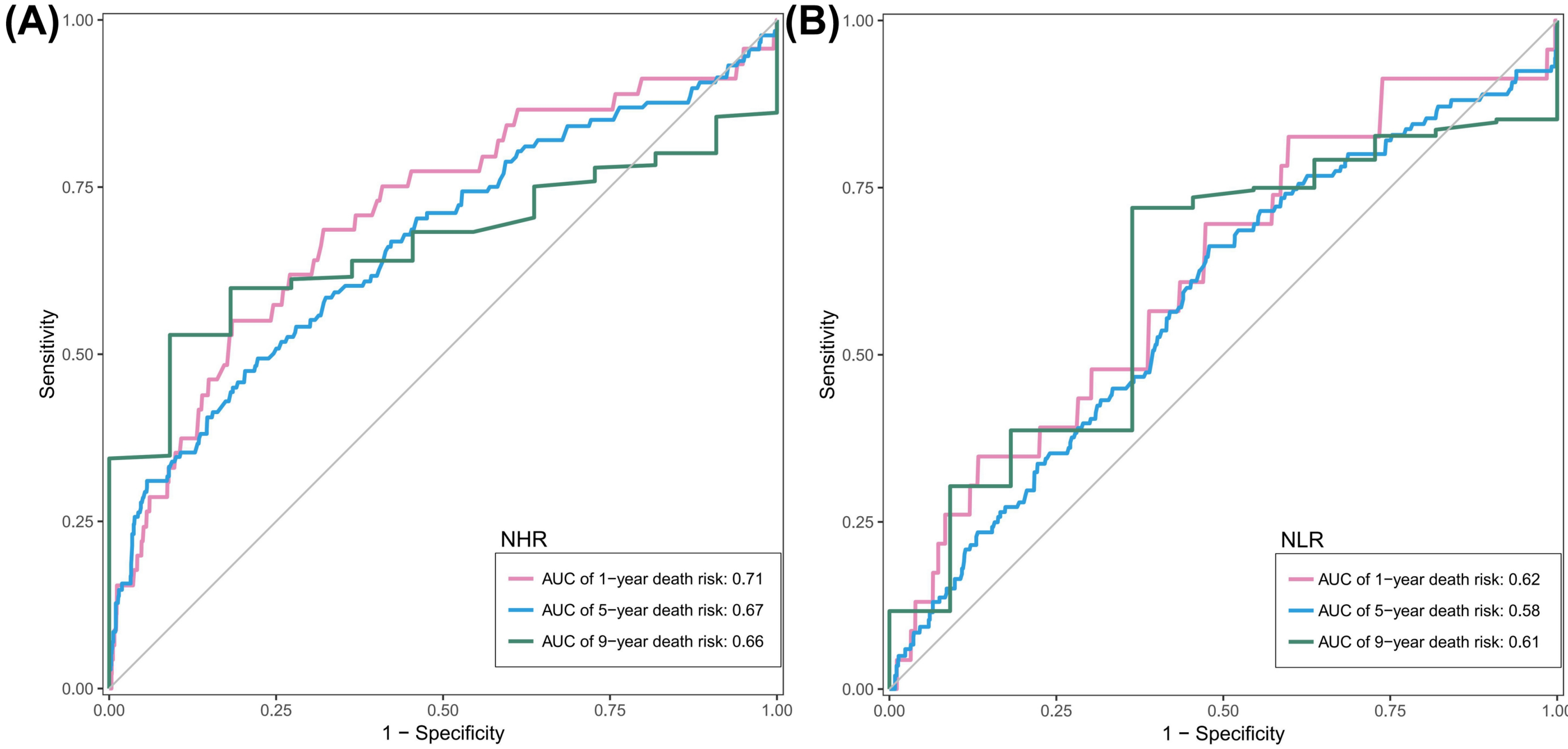

eFigure 4. Comparison of time-dependent ROC curves between NHR and NLR for predicting all-cause mortality among participants with asthma. Time-dependent ROC curves were generated to compare the discriminatory performance of NHR and NLR for 1-, 5-, and 9-year all-cause mortality.

Supplementary explanation of statistical methods

To ensure sample representativeness, appropriate weights were applied to account for the complex sampling design of NHANES. Sample weights for analysis were calculated as fasting sample 2-year MEC weight divided by 4. Statistical analyses were carried out using the chi-square test for categorical information and the Kruskal-Wallis rank-sum test for continuous information. To examine the association between mortality risk in asthmatics and NHR, three Cox proportional hazards regression models (Models I, II , and III) were employed. Model I adjusted for no covariates. Model I provided an unadjusted estimate, establishing the baseline association between NHR and mortality in asthmatic populations. Model II adjusted for age, race, and sex, which are widely recognized as fundamental confounders in epidemiological research, especially in studies of respiratory and metabolic diseases. These variables were selected because they are known to influence both the NHR and mortality in asthmatic populations. By adjusting for these basic demographic factors in Model II, we aimed to assess their specific impact on the association before proceeding to a more comprehensive adjustment. In the Model III, we adjusted covariates (race, age, sex, smoking, alcohol, education, marriage, BMI, GCS, hypertension, diabetes, CVD, cancer, other CAD [emphysema, chronic bronchitis, or COPD], hay fever, STC, STG and blood eosinophil count) that satisfied any one of the following criteria: (1) We controlled for a variable that would change the effect estimate by at least 10% if it was added to the model. (2) We also picked variables based on previous research and the database's limitations. (3) The multicollinearity of the variables was evaluated using variance inflation factor (VIF) values. All covariates included in Model III have a VIF less than 5. Covariates with VIF greater than 5 indicate multicollinearity with the independent variable NHR, and these covariates were excluded. Restricted cubic splines (RCS) was then utilized to further quantify this relationship, adjusting for all covariates. The

cumulative death risk curve, complemented by survival 3D plot, quantified the impact of NHR on survive outcomes throughout the follow-up period. Employing the Least Absolute Shrinkage and Selection Operator (LASSO) regression model, we discerned the primary predictors of death risk while accounting for the covariation among the covariates. LASSO improved the accuracy and clarity of statistical models by adding a penalty to the regression equation based on the total of the absolute values of the coefficients. This model effectively eliminates variables with zero coefficients using the ten-fold cross-validation method with LassoCV. Using the optimal penalty parameter (lambda.1se), the following nine variables were selected: neutrophil to NHR, age, hypertension, diabetes, CVD, cancer, other CAD, GCS, and STC. These variables had non-zero coefficients at lambda.1se, reflecting a balance between model simplicity and predictive accuracy. These nine variables were then incorporated into the time-dependent receiver operating characteristic (ROC) curves to construct the final prediction model. The SHAP model was subsequently applied to determine the relative importance of variables in predicting mortality risk among asthma patients. To reduce the risk of overestimating model performance, we performed internal validation using 1,000 bootstrap resamples and 10-fold cross-validation. Bootstrap validation was used to evaluate the stability of time-dependent AUC estimates, and 10-fold cross-validation was used to assess the discriminatory performance of the model across resampled datasets. Time-dependent AUC values were calculated for 1-, 5-, and 9-year all-cause mortality. Missing covariates (smoking, alcohol, education, marriage, BMI, hypertension, diabetes, GCS, CVD, other CAD, hay fever, cancer, STC, STG, and blood eosinophil count), each constituting less than 10% of the total data, were managed through multiple imputations. Sensitivity analyses were conducted on the primary findings using the imputed dataset. This study applied the R 4.4.1 to carry out a series of statistical analyses. The ***P*** value < 0.05 implied the statistical difference. The core R packages used in this study included the “mice” package, "timeROC" package,"glmnet" package, "shapviz" package, "riskRegression" package, “contsurvplot” package, “plotRCS” package, etc.
